# Volatile organic compounds associated with red-stripe pathogens inhibit mycelial growth of *Morchella sextelata* by disrupting membrane integrity and inducing oxidative stress

**DOI:** 10.1128/spectrum.03958-25

**Published:** 2026-06-09

**Authors:** Xuetai Zhu, Yongxiang Dang, Qi Yang, Yaqin Zeng, Fei Su

**Affiliations:** 1 College of Life Sciences, Northwest Normal University, Lanzhou, China; Agroscope, Nyon, Switzerland

**Keywords:** *Bacillus subtilis*, *Pseudomonas chlororaphis*, antifungal mechanism, membrane integrity, oxidative stress

## Abstract

The commercial cultivation of *Morchella sextelata* is severely threatened by red-stripe disease, caused by the pathogenic bacteria, with *Bacillus subtilis* M-5 and *Pseudomonas chlororaphis* subsp. *aureofaciens* M-B being among the causative agents. This study revealed that volatile organic compounds (VOCs) emitted by these pathogens completely inhibited the mycelial growth of *M. sextelata*, with scanning electron microscopy revealing severe hyphal shrinkage and collapse. Headspace-gas chromatography-ion mobility spectrometry identified 2,3-butanedione and 2-methylpropanoic acid as the predominant antifungal VOCs from strains M-B and M-5, respectively. Exogenous application confirmed their potent inhibitory activity, with minimum inhibitory concentrations (MIC) of 150 µL/mL and 250 µL/mL. Mechanistic investigations demonstrated that these VOCs, particularly 2,3-butanedione and 2-methylpropanoic acid, disrupt cell membrane integrity, leading to significant leakage of intracellular nucleic acids and proteins, inducing lipid peroxidation, and triggering an antioxidant defense response characterized by elevated superoxide dismutase and peroxidase activities. Our findings elucidate that bacterial VOCs are volatile pathogenic factors responsible for growth arrest in red-stripe disease, providing a crucial theoretical foundation for developing novel management strategies for this devastating disease.

**IMPORTANCE:**

The cultivation of morels, particularly *Morchella sextelata*, is significantly constrained by the emergent red-stripe disease, yet effective and environmentally friendly control strategies are lacking. This study reveals a previously underappreciated mechanism of pathogenesis: VOCs produced by the causative bacteria, *Bacillus subtilis* M-5 and *Pseudomonas chlororaphis* subsp. *aureofaciens* M-B. We identify 2,3-butanedione and 2-methylpropanoic acid as key antifungal VOCs that exerts its effect by critically damaging hyphal membrane integrity, inducing the leakage of cellular contents, and disrupting redox homeostasis. These findings shift the conventional perspective on bacterial-fungal interactions in morel disease and highlight the critical role of pathogen-derived volatiles. Consequently, our work provides a direct theoretical foundation for optimizing ventilation management in greenhouse cultivation to disperse these harmful VOCs. This insight is pivotal for developing novel, green prevention tactics and ensuring the sustainable and productive cultivation of morels.

## INTRODUCTION

Morels have long been valued in gourmet cuisine for their uncommon amino acids and complex volatile aroma profile (1, 2). Recent studies have demonstrated that their polysaccharides possess immunomodulatory, antitumor, and antioxidant properties, establishing morel species as a priority resource for functional food development (3–7).

Since the successful establishment of commercial cultivation in China in 2010, morel production has expanded significantly (8, 9). However, the industry now faces a critical bottleneck: recurrent outbreaks of red-stripe disease, which often leads to severe yield losses or complete crop failure. During the disease outbreak, the stipe of already-formed *Morchella* fruiting bodies appears reddish-brown, after which the color gradually deepens and spreads from the base upward. The entire fruiting body turns red and emits a foul odor. As the disease progresses, the morel gradually loses vitality, experiences growth arrest, and eventually undergoes tissue rot, with no new fruiting bodies being produced. It spreads rapidly in the field, capable of devastating entire plantings. Published studies have identified the soil-borne bacteria *Bacillus subtilis* and *Pseudomonas chlororaphis* subsp. *aureofaciens* as the primary pathogens. Pathogenicity tests were subsequently conducted, and the experimental results demonstrated that the colonization of pathogenic bacteria caused the morel to become diseased (10).

Under our preliminary dual-plate assays, the radial growth of *M. sextelata* was significantly inhibited even when the bacterial pathogen was positioned more than 3 cm away from the mycelial inoculum. This observation suggests that VOCs released by the bacteria mediate this antagonistic effect. This finding also explains why, during outbreaks of red-stipe disease, *Morchella* ceases the production of new fruiting bodies while the existing ones turn red and undergo rot—because mycelial growth can be inhibited by bacterial VOCs without direct physical contact with the pathogen. This observation is consistent with established roles of bacterial VOCs in suppressing diverse fungal pathogens (11–14). For instance, Massawe et al. reported that eight VOCs produced by *Bacillus* spp. effectively inhibited sclerotial formation and hyphal extension in *Sclerotinia sclerotiorum* (15). Similarly, VOCs from *B. pumilus* and *B. thuringiensis* significantly restricted the *in vitro* colony expansion of *Colletotrichum gloeosporioides* (16). Mechanistically, such volatiles can directly disrupt fungal cell wall and membrane integrity, inducing leakage of intracellular components (17–21); a representative example is 2,3-butanedione from *B. subtilis* CL2, which damages the membrane of *Aspergillus flavus* (22). Additionally, VOCs can provoke reactive oxygen species (ROS) bursts and disrupt cellular redox homeostasis, ultimately triggering apoptosis or cell death. Supporting this, VOCs derived from *Pseudomonas chlororaphis* subsp. *aureofaciens* SPS-41 were shown to induce ROS accumulation and aberrantly elevate catalase (CAT) and superoxide dismutase (SOD) activities in *Ceratocystis fimbriata*, leading to hyphal growth inhibition (23). VOCs released by *Stenotrophomonas* sp. NAU1697 significantly inhibit mycelial elongation and spore germination in *Fusarium oxysporum* f. sp. *cucumerinum*, with 2-ethylhexanol showing the highest activity. Following treatment, the integrity of the mycelial cell membrane was compromised, ergosterol levels were decreased, and a ROS burst was observed (24).

The volatile-mediated antagonism of *B. subtilis* and *P. chlororaphis* subsp. *aureofaciens* against *M. sextelata* has not been systematically elucidated. Consequently, this study was designed to (i) verify the inhibitory effects of VOCs emitted by these pathogens on the hyphal growth of *M. sextelata*, (ii) characterize the volatile blends via GC–MS and pinpoint the bioactive constituents through exogenous application assays, and (iii) investigate the mechanistic basis of inhibition by assessing mycelial membrane integrity, intracellular ROS accumulation, and key antioxidant enzyme activities. Our findings are expected to delineate the functional role of bacterial VOCs in this pathosystem, thereby providing a theoretical foundation for developing novel biocontrol strategies against red-stripe disease and addressing a critical knowledge gap in fungus–bacteria interactions within the genus *Morchella*.

## MATERIALS AND METHODS

### Plate confrontation assay for evaluating the inhibitory effects of VOCs on mycelial growth

A plate confrontation assay was conducted to evaluate the inhibitory effects of VOCs emitted by *Bacillus subtilis* M-5 and *Pseudomonas chlororaphis* subsp. *aureofaciens* M-B on the mycelial growth of three commercial cultivated *Morchella* species: *M. sextelata* M6611, *M. importuna*, and *M. septimelata*. The bacterial strains, previously identified as pathogens associated with *M. sextelata* red-stripe disease, were pre-cultured in liquid LB medium at 28°C with shaking at 180 rpm for 24 h. Subsequently, 100 µL of each bacterial suspension (approximately 1  ×  10^8^ CFU/mL) was spread evenly onto individual LB agar plates.

A mycelial plug (8 mm in diameter), taken from the actively growing margin of a fungal colony, was placed at the center of a fresh potato dextrose agar (PDA) plate. The PDA plate inoculated with the fungal plug was then inverted and placed over the LB agar plate spread with the bacterial suspension to create a dual-culture assembly, allowing VOC exposure without physical contact. The two plates were securely sealed together with Parafilm. In the control group, the fungal-inoculated PDA plate was paired with a blank LB plate (without bacteria). All treatments and the control were performed in three independent biological replicates.

The assembled plates were incubated at 15°C for 5 days. After incubation, the diameter of the fungal colonies was measured using a vernier caliper. The percentage of mycelial growth inhibition (MGI) was calculated according to the following formula: MGI (%) = [(Dc – Dt) / Dc] × 100, where Dc represents the average colony diameter in the control group and Dt represents the average colony diameter in the treatment group (25).

### Scanning electron microscopy of *M. sextelata* M6611 mycelium

When the mycelia of *M. sextelata* M6611 grew to 1/5 of the PDA plate, *B. subtilis* M-5 and *P. chlororaphis* subsp. *aureofaciens* M-B, which had been pre-cultured in LB liquid medium at 28℃ and 180 rpm for 24 h prior to the experiment, were collected. Then, 100 μL of bacterial suspension (approximately 1 × 10⁸ CFU/mL) was evenly spread onto LB solid plates and subjected to plate confrontation with *M. sextelata* M6611. Subsequently, the co-culture device was incubated at 15℃ for 5 days.

To observe the morphological alterations in the mycelia induced by the fumigation treatment, scanning electron microscopy (SEM) analysis was performed. Mycelial samples of *M. sextelata* M6611 were fixed overnight at 4°C in 2.5% (vol/vol) glutaraldehyde, followed by post-fixation with 1% (wt/vol) osmium tetroxide for 1–2 h. The fixed samples were then dehydrated through a graded ethanol series (30%, 50%, 70%, 85%, 95%, and 100%), subjected to critical-point drying, and mounted on aluminum stubs using conductive adhesive. Subsequently, the samples were sputter-coated with a thin layer of gold-palladium (26). The morphological characteristics of the prepared mycelia were examined using a scanning electron microscope (JEOL 7610PLUS) at an accelerating voltage of 10 kV.

### Analysis of VOCs from strains M-5 and M-B

The VOCs of strains M-5 and M-B were characterized to identify specific metabolites potentially responsible for the observed antifungal activity. VOCs collection and analysis were performed using headspace-gas chromatography-ion mobility spectrometry (HS-GC-IMS). Briefly, M-5 and M-B were pre-cultured to a density of 10^8^ CFU/mL, determined by optical density measurement. A 100 μL aliquot of each bacterial suspension was inoculated into a 20 mL culture flask containing 5 mL of LB broth. An uninoculated LB broth flask was prepared under identical conditions to serve as the negative control. All flasks were sealed and incubated at 28°C for 3 days (27).

After cultivation, headspace gas from each conical flask was sampled for analysis. The incubation temperature was 50°C; after 15 min of incubation, a 500 µL sample was injected in splitless mode. The incubation shaking speed was 500 r/min, and the syringe temperature was 85°C. GC conditions: the column temperature was 60°C; the carrier gas was high-purity nitrogen (purity ≥ 99.999%); the programed pressure increase was as follows: initial flow rate of 2.0 mL/min held for 2 min, linearly increased to 10.0 mL/min over 8 min, and then linearly increased to 100.0 mL/min over 10 min. The chromatographic run time was 20 min, and the injection port temperature was 80°C. IMS conditions: the ionization source was a tritium source (³H); the migration tube length was 53 mm; the electric field strength was 500 V/cm; the migration tube temperature was 45°C; the drift gas was high-purity nitrogen (purity ≥ 99.999%) with a flow rate of 150 mL/min; positive ion mode was used. VOCs were identified by comparing the obtained spectra with the NIST 2020 mass spectrometry library and cross-referencing with the IMS migration database. Each experimental treatment group and control group was independently repeated three times (*n* = 3).

### Assessment of mycelial growth inhibition by pure synthetic VOCs

The inhibitory effects of pure synthetic volatile compounds on *M. sextelata* M6611 were evaluated using a sealed-plate assay. The synthetic compounds were procured from Tianqi Gene Co., Ltd. Mycelial plugs (8 mm in diameter) of *M. sextelata* M6611 were placed at the center of fresh PDA plates. Subsequently, 10 µL of each pure synthetic compound (500 µL/mL) was applied to a sterile filter paper disc (8 mm diameter), which was then affixed to the inner center of the Petri dish lid. The plates were immediately sealed with parafilm to create a sealed atmosphere, thereby allowing volatile exposure to the mycelium, and incubated at 15°C for 5 days. The inhibition rate of mycelial growth was calculated for each compound after the incubation period.

The MIC of the most effective volatile compounds was determined according to the method described by Zhou et al. (28) Briefly, a two-sealed-plate assembly was used, wherein the volatile compound was applied at a series of graded concentrations to the lid compartment, and the fungal mycelium was inoculated on the PDA medium in the base compartment. The MIC was defined as the lowest concentration that completely suppressed visible mycelial growth after incubation.

### Investigation of the inhibition mechanism of 2,3-butanedione and 2-methylpropanoic acid on *M. sextelata* M6611

#### Intracellular substance leakage assay

Given that 2,3-butanedione and 2-methylpropanoic acid showed the most pronounced inhibitory effects from M-5 and M-B, we assessed intracellular substance leakage to determine whether they disrupt fungal cell membrane integrity. Mycelial plugs of *M. sextelata* M6611 were inoculated at the center of PDA agar plates and incubated under appropriate conditions. Various concentrations of 2,3-butanedione (1/2 MIC: 75 μL/mL, MIC: 150 μL/mL, 2 MIC: 300 μL/mL) and 2-methylpropanoic acid (1/2 MIC: 125 μL/mL, MIC: 250 μL/mL, 2 MIC: 500 μL/mL) were applied as fumigation treatments, with untreated mycelial cultures serving as the control. After treatment, mycelial samples were harvested and centrifuged at 8,000 × *g* for 5 min. The absorbance of the resulting supernatant was measured at 260 nm and 280 nm to assess nucleic acid and protein leakage, respectively. All experiments were performed in triplicate (29).

#### Malondialdehyde determination

The extent of lipid peroxidation, an indicator of oxidative damage to cell membranes, was evaluated by measuring the MDA content. The MDA content was determined according to the method described by Zhao et al. (30). Briefly, 0.1 g of mycelial samples was homogenized in a pre-cooled mortar with a small amount of quartz sand and 2 mL of 10% (wt/vol) trichloroacetic acid (TCA). The homogenate was centrifuged at 12,000 × *g* for 10 min at 4°C. Then, 0.4 mL of the supernatant was mixed thoroughly with 0.4 mL of 0.6% thiobarbituric acid (TBA) solution. The mixture was incubated in a boiling water bath for 15 min, cooled to room temperature, and centrifuged again. The absorbance of the supernatant was measured at 450 nm, 532 nm, and 600 nm.

#### SOD and peroxidase determination

To investigate the antioxidant response of *M. sextelata* M6611 to 2,3-butanedione and 2-methylpropanoic acid induced oxidative stress, the activities of SOD and POD were measured. Mycelial samples were collected, and the activities of SOD and POD were measured using commercial assay kits (AIDISHENG, China) according to the manufacturer’s instructions. Each assay was performed in three independent biological replicates to ensure statistical reliability.

## RESULTS

### Inhibitory effects of VOCs produced by M-5 and M-B on mycelial growth

**Fig 1:**
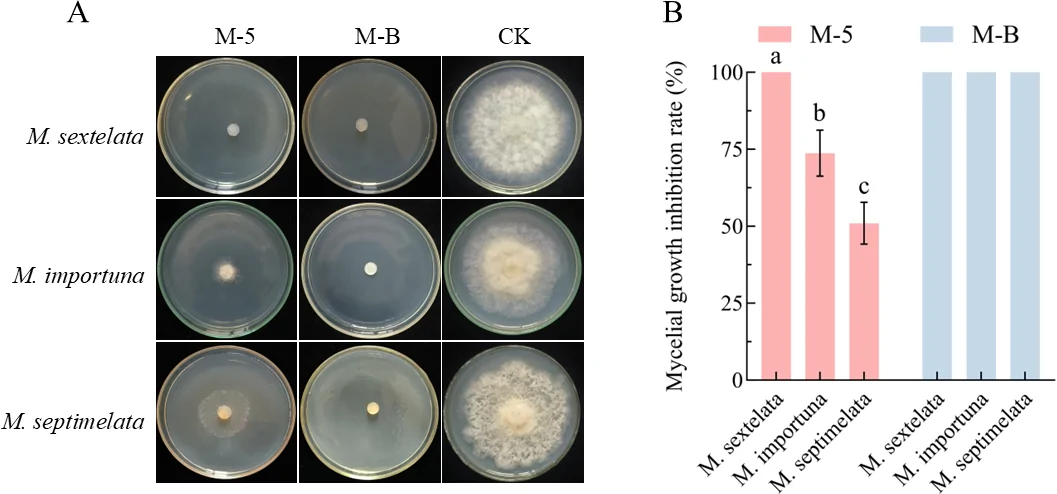
(**A and B**) Inhibitory effects of VOCs produced by M-5 and M-B on mycelial growth.

The VOCs emitted by strains M-5 and M-B exhibited significant inhibitory effects on the mycelial growth of three *Morchella* species: *M. sextelata* M6611, *M. importuna*, and *M. septimelata* (Fig. 1). The most pronounced inhibition was observed against the host fungus, *M. sextelata* M6611, where its mycelial growth was completely suppressed by the VOCs from both bacterial strains.

### Morphological alterations in *M. sextelata* M6611 myceliuminduced by bacterial VOCs

**Fig 2:**
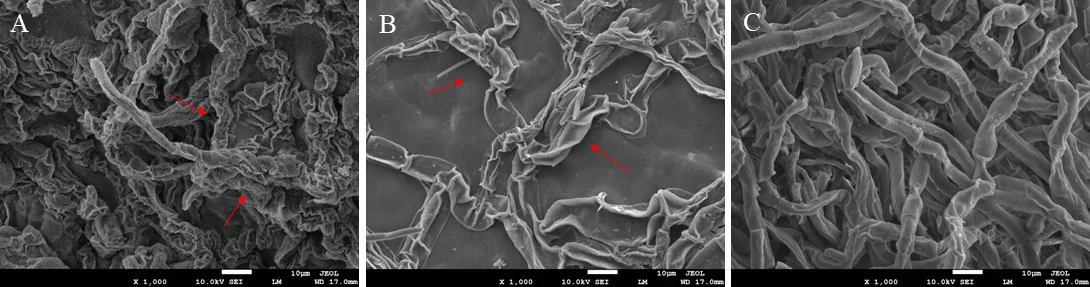
Effects of bacterial VOCs on the mycelial structure of *M. sextelata* M6611. Red arrows highlight morphological abnormalities in VOC-treated groups: (**A**) M-5 and (**B**) M-B, compared with (**C**) the normal hyphae in the control (CK).

SEM revealed distinct morphological alterations in the mycelium of *M. sextelata* M6611 following exposure to VOCs from strains M-5 and M-B (Fig. 2). In the control group, the mycelia displayed a robust, well-defined, and uniform structure with an intact surface. In contrast, treatment with VOCs from M-5 disrupted the linear hyphal morphology, resulting in an irregular diameter and the formation of verrucose protuberances. The VOCs emitted by M-B induced more severe damage, causing the hyphae to become notably twisted, shriveled, and ultimately leading to cellular collapse and rupture.

### Identification of VOCs produced by M-5 and M-B

**Fig 3:**
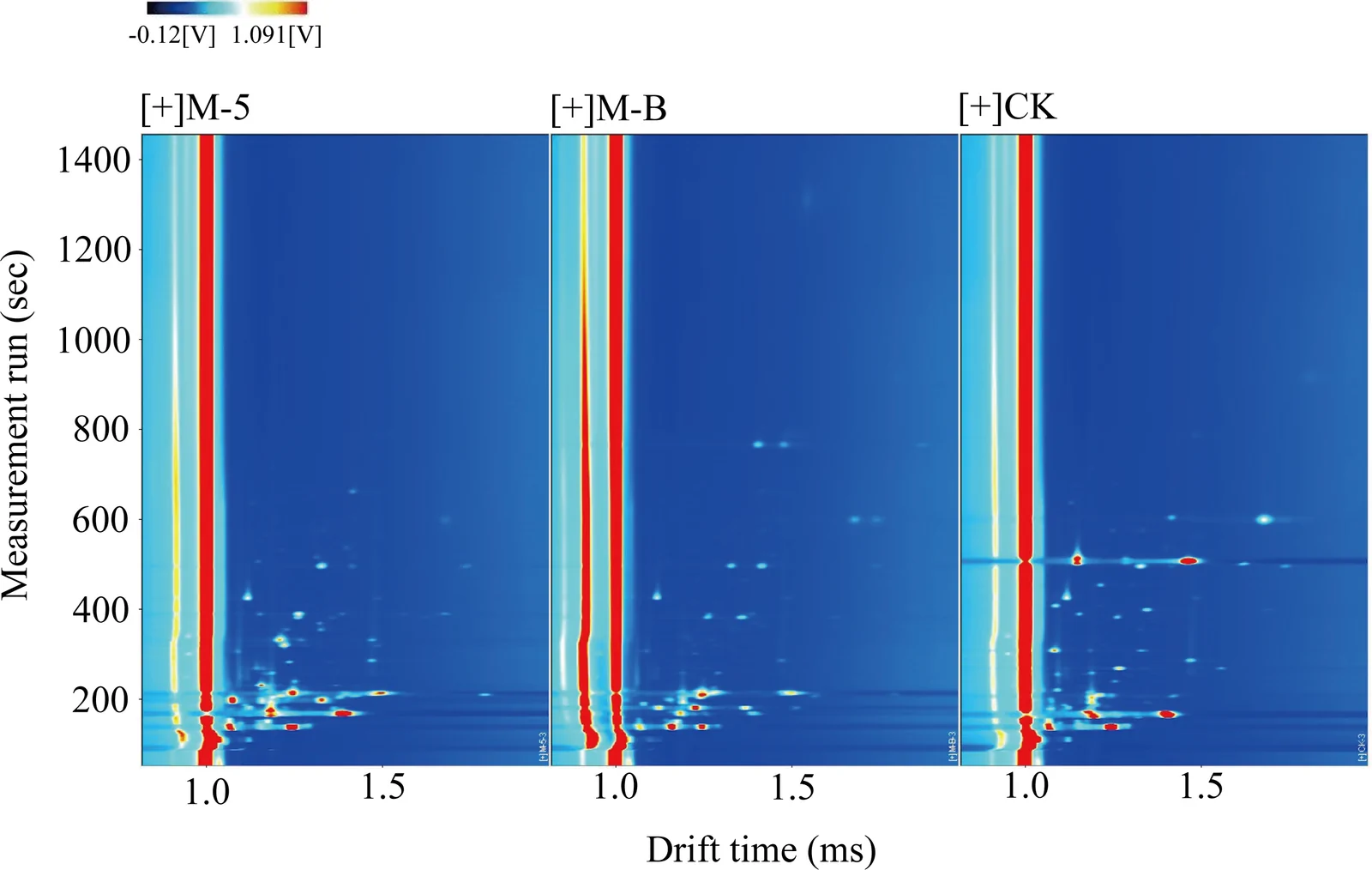
GC-IMS spectrum (top view).

**Fig 4:**
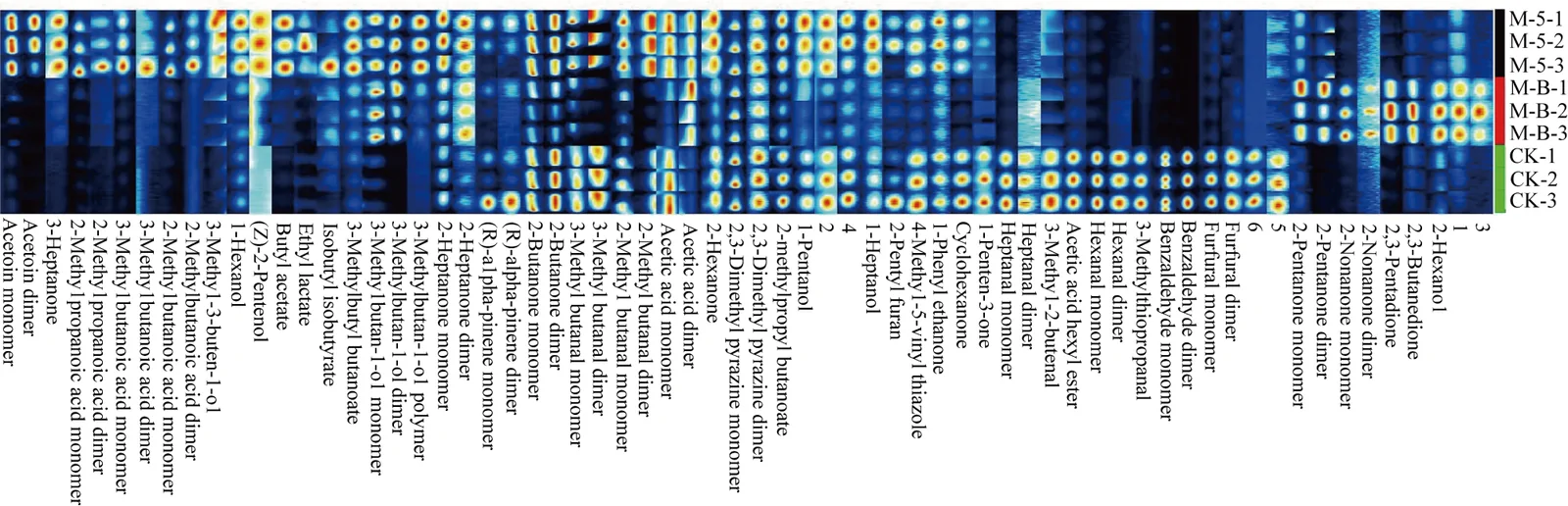
Comparative GC-IMS fingerprint of VOCs from M-5 and M-B. Each row corresponds to the complete VOC profile of a single sample, while each column aligns the signal peaks of the same VOC across different samples. Numeric codes denote unidentified compounds.

The VOCs profiles of strains M-5 and M-B were characterized using HS-GC-IMS. The topographic plots (Fig. 3) revealed distinct differences in VOC composition and concentration among the samples, with the majority of compounds eluting before 800 s and exhibiting drift times between 1.0 and 1.75 ms. A total of 67 signal peaks, corresponding to identified and unidentified VOCs, were detected in the comparative fingerprint (Fig. 4). Specifically, HS-GC-IMS analysis identified 13 VOCs in M-5, including cetoin, 2-methylpropanoic acid, and 3-methylbutanoic acid, while M-B produced 5 VOCs, such as 2,3-butanedione and 2,3-pentadione (Table 1).

**TABLE 1:** VOCs identified in strains M-5 and M-B by HS-GC-IMS

Count	Compound	CAS#	Formula	MW	RI	Rt [sec]	Dt [RIPrel]
VOCs in M-5							
1	Acetoin	C513860	C_4_H_8_O_2_	88.1	711	198.866	1.32944
2	3-Heptanone	C106354	C_7_H_14_O	114.2	871.7	356.198	1.23076
3	2-Methylpropanoic acid	C79312	C_4_H_8_O_2_	88.1	752.5	231.994	1.37272
4	3-Methylbutanoic acid	C503742	C_5_H_10_O_2_	102.1	841.8	320.243	1.22365
5	3-Methyl-3-buten-1-ol	C763326	C_5_H_10_O	86.1	743.1	224.072	1.15913
6	1-Hexanol	C111273	C_6_H_14_O	102.2	871.4	355.792	1.32438
7	(Z)−2-Pentenol	C1576950	C_5_H_10_O	86.1	772.4	249.79	0.94159
8	Butyl acetate	C123864	C_6_H_12_O_2_	116.2	807.4	283.485	1.23591
9	Ethyl lactate	C97643	C_5_H_10_O_3_	118.1	793.9	270.162	1.14517
10	Isobutyl isobutyrate	C97858	C_8_H_16_O_2_	144.2	882.9	370.623	1.32484
11	3-Methylbutyl butanoate	C106274	C_9_H_18_O_2_	158.2	1047.1	663.857	1.41888
12	3-Methylbutan-1-ol	C123513	C_5_H_12_O	88.1	729.4	212.909	1.24708
13	2-Heptanone	C110430	C_7_H_14_O	114.2	892	382.915	1.26062
VOCs in M-B							
14	2-Hexanol	C626937	C_6_H_14_O	102.2	782.9	259.719	1.29035
15	2, 3-Butanedione	C431038	C_4_H_6_O_2_	86.1	590.8	140.568	1.15829
16	2, 3-Pentadione	C600146	C_5_H_8_O_2_	100.1	686.5	181.943	1.22893
17	2-Nonanone	C821556	C_9_H_18_O	142.2	1096.7	765.36	1.40329
18	2-Pentanone	C107879	C_5_H_10_O	86.1	689.2	183.383	1.12283

### Inhibition of the *M. sextelata* M6611 by pure synthetic VOC components

**Fig 5:**
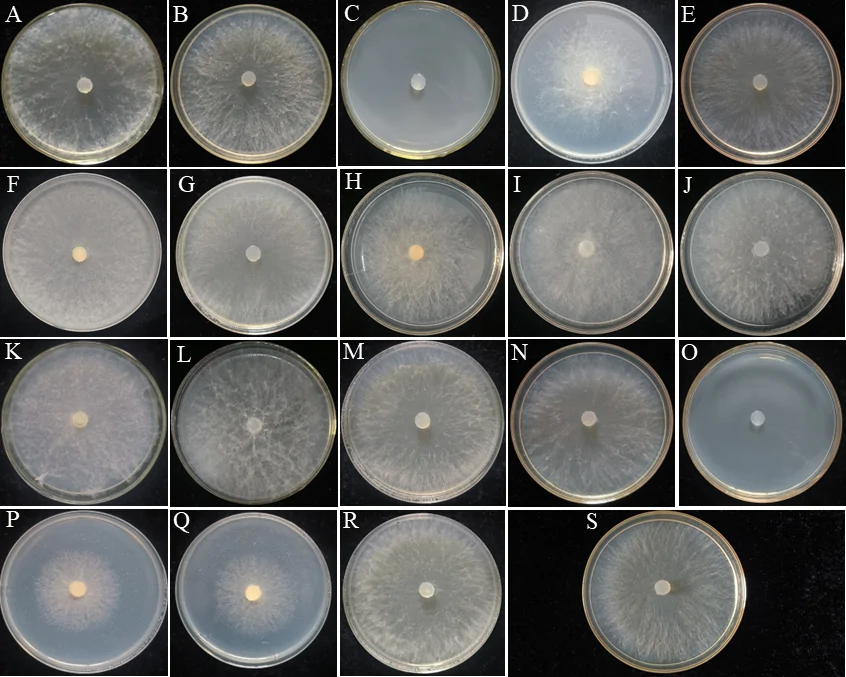
Inhibition of *M. sextelata* M6611 mycelial growth by synthetic VOC components. The assay evaluated various volatile organic compounds identified from strains M-5 and M-B. Letters A to S correspond to specific compounds listed in the figure annotations. Note the differential efficacy among treatments, with compounds C (2-methylpropanoic acid) and O (2,3-butanedione) showing pronounced inhibition compared to the control (S, CK).

**Fig 6:**
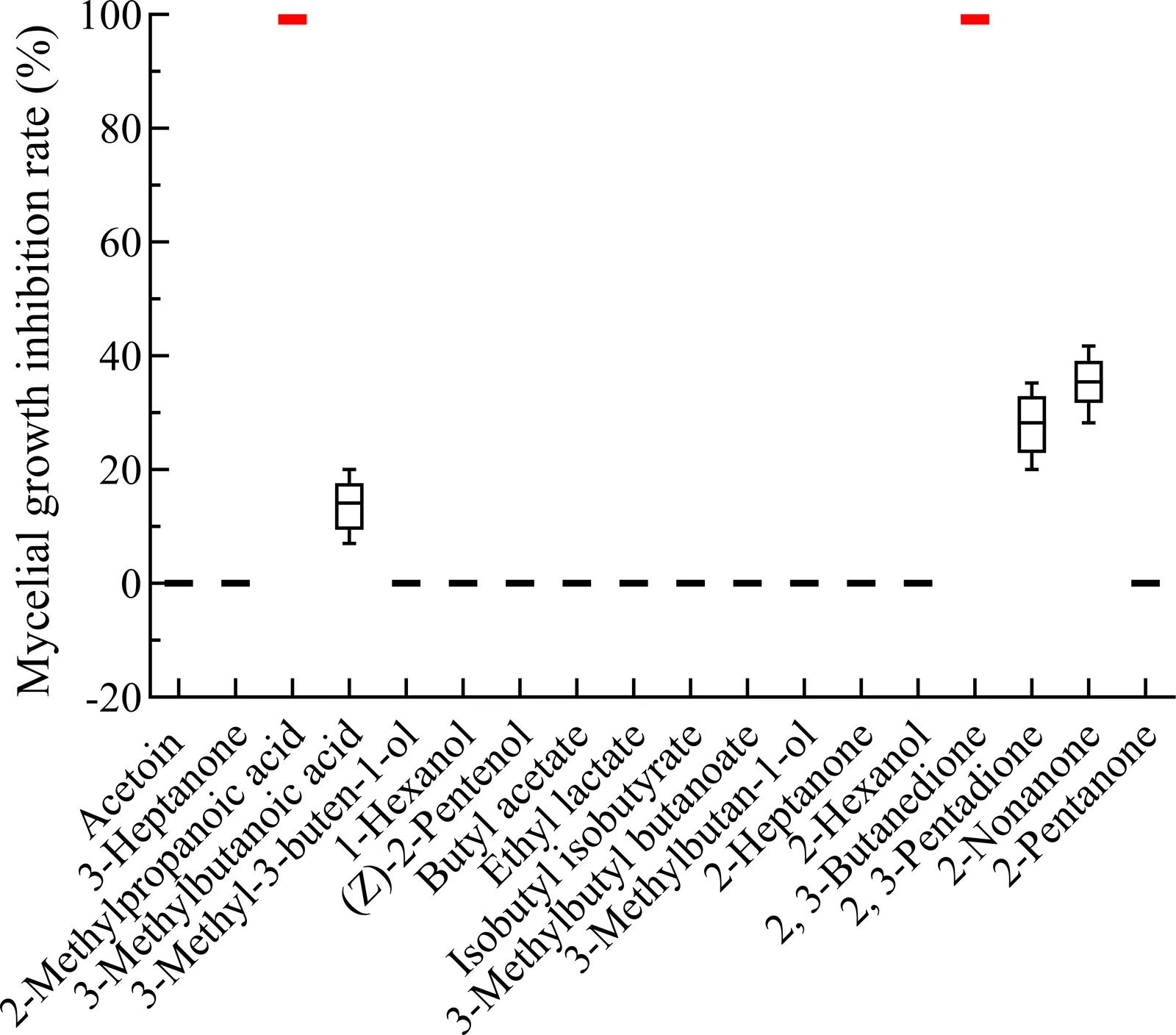
Mycelial growth inhibition rate of *M. sextelata* M6611 treated with synthetic VOC components from M-5 and M-B. The bar graph quantifies the inhibitory efficacy of each individual volatile compound compared to the untreated control (CK). Compounds 2,3-butanedione and 2-methylpropanoic acid exhibited the highest inhibition rates.

The inhibitory effects of volatile organic compounds on the mycelial growth of *M. sextelata* M6611 were assessed. Among the compounds tested, 2-methylpropanoic acid and 2,3-butanedione demonstrated potent inhibition (Fig. 5 and 6). The MIC of 2-Methylpropanoic acid was determined to be 250 μL/mL, while that of 2,3-butanedione was 150 μL/mL (Table 2). Based on the lower MIC value, 2,3-butanedione was identified as the most effective compound in suppressing the growth of *M. sextelata* M6611.

**TABLE 2:** Determination of the MIC of 2,3-butanedione and 2-methylpropanoic acid against *M. sextelata* M6611

Concentration (μL/mL)	Mycelial growth inhibition (%)	
	2-Methylpropanoic acid	2,3-Butanedione
50	0	15.47 ± 1.07
100	15.20 ± 1.36	54.04 ± 1.68
150	43.88 ± 2.43	100
200	74.88 ± 1.17	100
250	100	100
300	100	100
350	100	100
400	100	100
450	100	100
500	100	100

### Investigation of the inhibition mechanism of 2,3-butanedione and 2-methylpropanoic acid on *M. sextelata* M6611

#### Intracellular substance leakage assay

**Fig 7:**
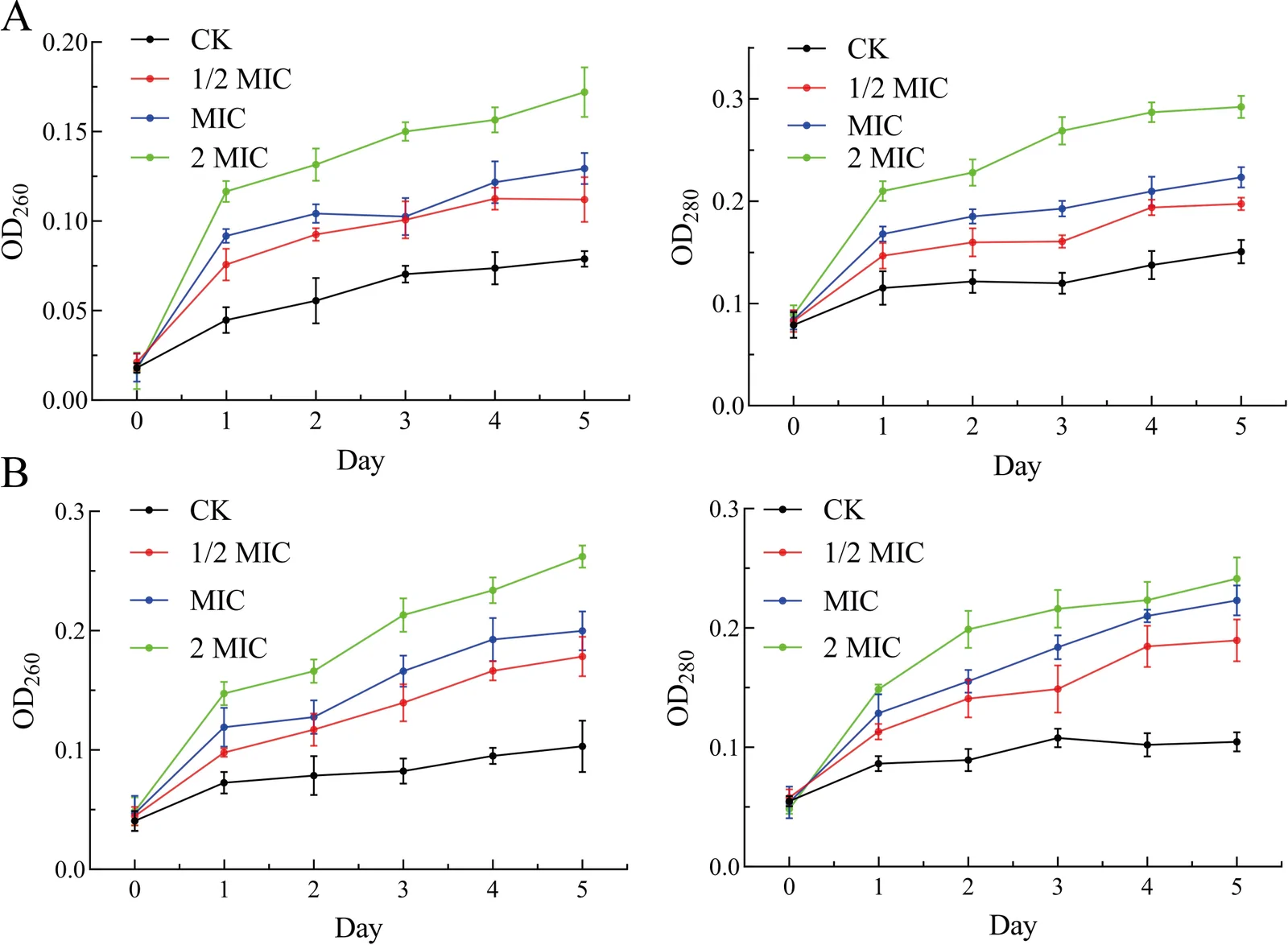
Effects of 2,3-butanedione and 2-methylpropanoic acid on plasma membrane integrity (**A**: 2,3-butanedione; **B**: 2-methylpropanoic acid).

Exposure to 2,3-butanedione and 2-methylpropanoic acid resulted in a significant, time-dependent increase in the leakage of nucleic acids and proteins from *M. sextelata* M6611 mycelia compared to the control (Fig. 7).

#### Lipid peroxidation assay (MDA content)

**Fig 8:**
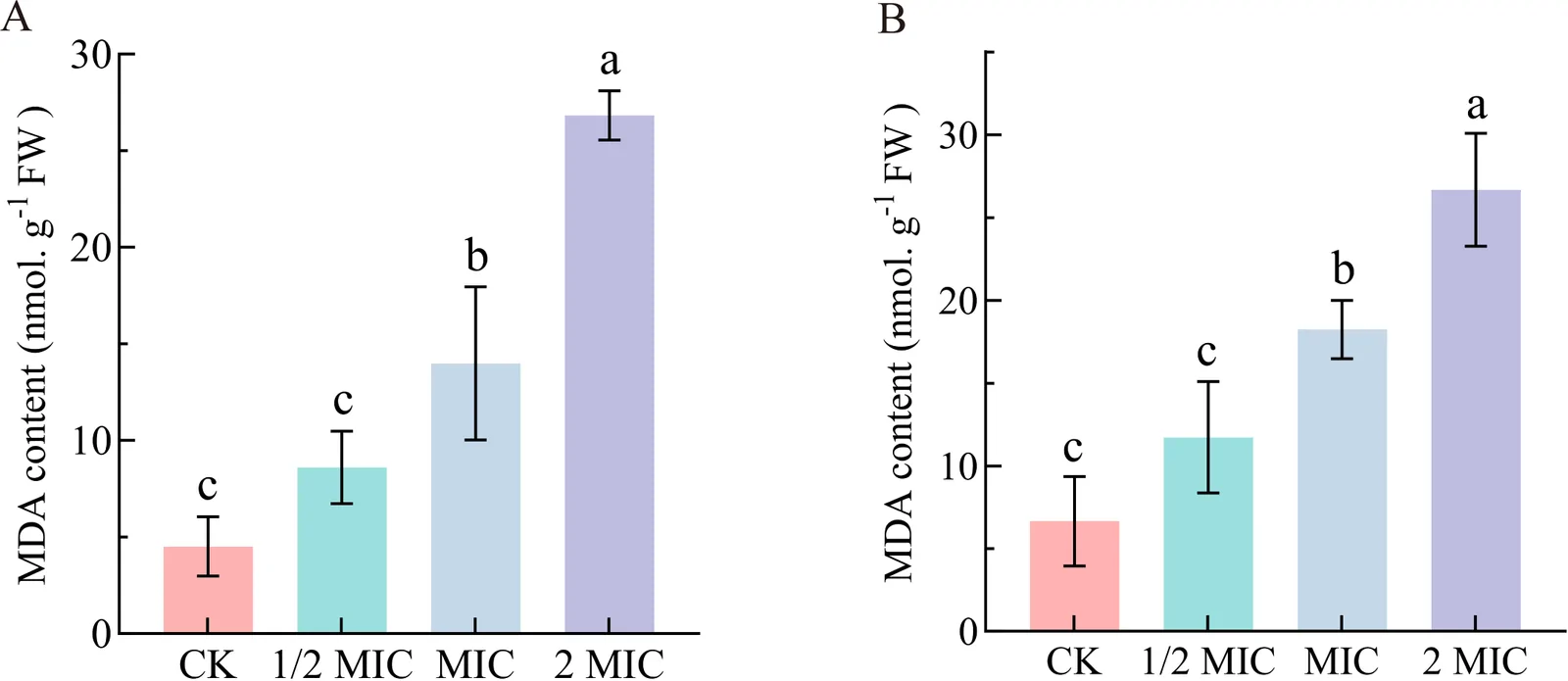
Effect of 2,3-butanedione and 2-methylpropanoic acid on MDA content in *M. sextelata* M6611. Data are shown as means ± SD (**A**: 2,3-butanedione; **B**: 2-methylpropanoic acid).

The level of MDA, a marker of lipid peroxidation, was significantly elevated in mycelia treated with either compound (Fig. 8). The MDA content increased with higher treatment concentrations, confirming that both 2,3-butanedione and 2-methylpropanoic acid induce oxidative damage to the cell membrane.

#### Antioxidant enzyme activities (SOD and POD)

**Fig 9:**
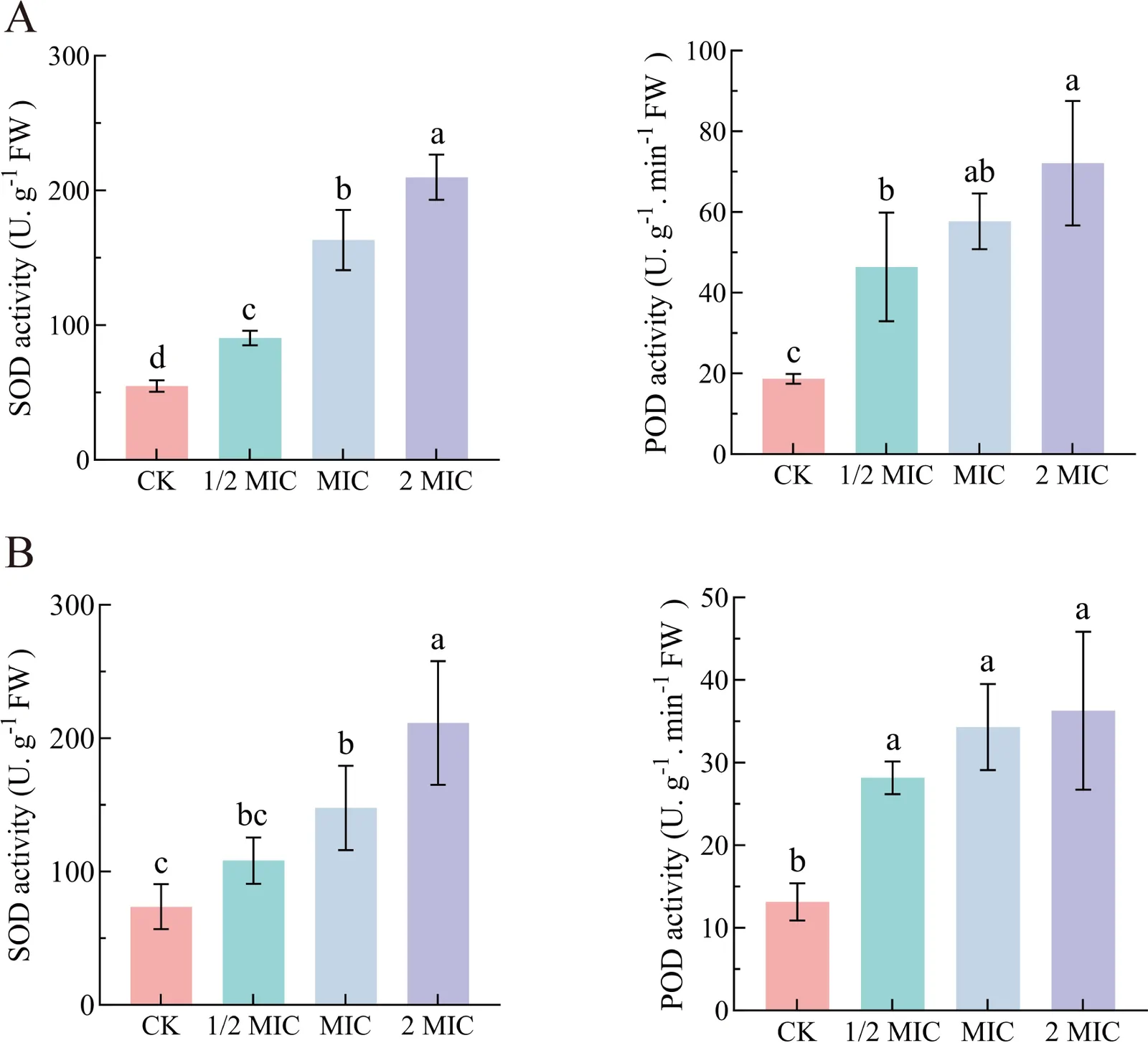
Effect of 2,3-butanedione and 2-methylpropanoic acid on antioxidant enzyme activities in *M. sextelata* M6611. Data are shown as means ± SD (**A**: 2,3-butanedione; **B**: 2-methylpropanoic acid).

The activities of the antioxidant enzymes SOD and POD were significantly upregulated in response to the oxidative stress induced by both compounds (Fig. 9). This concentration-dependent increase in enzyme activity signifies the activation of the cellular antioxidant defense system in *M. sextelata* M6611.

## DISCUSSION

Red-stripe disease, induced by pathogenic bacteria, has emerged as a major constraint in the commercial cultivation of *M. sextelata*. This study systematically investigated the antifungal activity and mechanisms of VOCs released by two causative agents, *Bacillus subtilis* M-5 and *Pseudomonas chlororaphis* subsp. *aureofaciens* M-B. Dual-culture assays demonstrated that VOCs from both strains completely inhibited mycelial growth of *M. sextelata*, with SEM further revealing severe hyphal shrinkage and structural collapse. HS-GC-IMS identified 2,3-butanedione and 2-methylpropanoic acid as the dominant antifungal volatiles, with 2,3-butanedione exhibiting the strongest activity. Further mechanistic investigations indicated that 2,3-butanedione and 2-methylpropanoic acid disrupt hyphal membrane integrity, causing the leakage of intracellular proteins and nucleic acids, and induces oxidative stress accompanied by the accumulation of reactive oxygen species. These findings elucidate a key pathway in the pathogenesis of red-stripe disease and underscore the significance of VOC-mediated inhibition.

2,3-Butanedione is a widely used butter flavoring agent in the food industry, and its antimicrobial properties have been confirmed for many years (31, 32). However, research on its antifungal properties and mechanisms of action is relatively limited. Guangjin Li et al. (33) found that 2,3-butanedione can effectively inhibit the growth of *Botrytis cinerea*. Transcriptomic analysis showed that after treatment with 2,3-butanedione, the expression of genes related to proteolysis, peroxisomes, and autophagy in *B. cinerea* was significantly increased. Peroxisomes have the function of decomposing ROS within cells (34, 35), so it is speculated that 2,3-butanedione may cause cell death by inducing the accumulation of ROS. Additionally, Ling et al. (36) found that treatment with 2,3-butanedione increased the leakage of substances that absorb at 260 and 280 nm in the Fungal pathogens causing postharvest fruit rot, indicating damage to the cell membrane. In this study, it was found that 2,3-butanedione can disrupt the integrity of the cell membrane of *M. sextelata* and interfere with its oxidative balance, leading to ROS accumulation and thus inhibiting the growth of *M. sextelata*. This mechanism is consistent with the inhibitory mechanisms of biocontrol bacterial VOCs against plant pathogenic fungi in current studies (37).

Notably, our agar plate confrontation tests revealed that compared to *M. sextelata*, other species such as *M. importuna* and *M. septimelata* exhibited a degree of resistance to the VOCs emitted by strains M-5 and M-B. This interspecific variation suggests a potential strategy for disease management: screening for resistant genotypes from less susceptible species and implementing crop rotation with *M. sextelata* could be a feasible approach to mitigate outbreaks of red-stripe disease. However, in this study, we have not yet systematically characterized the potential resistance traits of *M. importuna* and *M. septimelata*. Future work could focus on cell wall composition analysis, antioxidant enzyme activity assays, and transcriptomic comparisons to elucidate the differential response mechanisms of different Morchella species to bacterial VOCs.

Although the inhibitory effects of bacterial VOCs on phytopathogenic fungi are well documented (38, 39), their impact on edible mushrooms has received little attention. Here, we demonstrate that morel mycelium is highly sensitive to bacterial VOCs, offering a new perspective for crop management. Among greenhouse environmental factors, temperature, humidity, O₂, and CO₂ (9) levels have long been emphasized. However, poor ventilation is accompanied by elevated CO₂ levels and increased humidity, conditions that themselves may promote bacterial proliferation, leading to the massive propagation of the red-stripe disease pathogens *B. subtilis* M-5 and *P. chlororaphis* subsp. *aureofaciens* M-B, and consequently resulting in greater accumulation of VOCs. Therefore, enhanced ventilation management not only reduces environmental CO₂ levels and humidity but also effectively suppresses pathogen proliferation and their VOCs production. Collectively, this study not only provides a theoretical basis for the integrated and sustainable control of red-stripe disease but also underscores the importance of VOC monitoring and ventilation management for the healthy development of the morel industry.

## Data Availability

The data sets used and/or analyzed during the current study available from the corresponding author on reasonable request. The strains *B. subtilis* M-5, *P. chlororaphis* subsp. *aureofaciens* M-B, and *M. sextelata* M6611 are all preserved in the Macrofungal Resources and Taxonomy Research Laboratory, College of Life Sciences, Northwest Normal University.
